# 2213 Tumor suppressors p53 and ARF control oncogenic potential of triple-negative breast cancer cells by regulating RNA editing enzyme ADAR1

**DOI:** 10.1017/cts.2018.143

**Published:** 2018-11-21

**Authors:** Che-Pei Kung, Emily A. Bross, Catherine E. Kuzmicki, Michael Benjamin, Leonard B. Maggi, Jason D. Weber

**Affiliations:** 1 Washington University in St. Louis; 2 Institute of Clinical and Translational Sciences, Washington University in St. Louis

## Abstract

OBJECTIVES/SPECIFIC AIMS: Triple-negative breast cancer (TNBC) accounts for one-fifth of the breast cancer patient population. The heterogeneous nature of TNBC and lack of options for targeted therapy make its treatment a constant adventure. The deficiency of tumor suppressors p53 and ARF is one of the known genetic signatures enriched in TNBC. Crucial questions remain about how TNBC is regulated by these genetic alterations. METHODS/STUDY POPULATION: In order to address this issue, we established p53/ARF-defective murine embryonic fibroblast and mammary epithelial cell to study the molecular and phenotypic consequences. Moreover, transgenic mice were generated to investigate the effect of p53/ARF deficiency on mammary tumor development in vivo. RESULTS/ANTICIPATED RESULTS: Increased proliferation and transformation capability were observed in p53/ARF-defective cells, and an aggressive form of mammary tumor was also seen in p53^−/−^ARF^−/−^ mice. Gene expression profiling and knock-down experiments using shRNAs were conducted to identify inflammatory marker ISG15 and RNA-editing enzyme ADAR1 as potential culprits for the elevated oncogenic potential. Interestingly, we found that the overexpression of ISG15 and ADAR1 is also prevalent in human TNBC cell lines. Reducing ADAR1 expression abrogated the oncogenic potential of human TNBC cell lines, while non-TNBC cells are less susceptible. DISCUSSION/SIGNIFICANCE OF IMPACT: These results indicate critical roles played by the tumor suppressors p53 and ARF in the pathogenesis of TNBC, likely through regulating ADAR1-mediated RNA modifications. Further understanding of this pathway promises to shed light on genetics-driven vulnerabilities of TNBC and inform development of more effective therapeutic strategies.

